# Type 3c Diabetes Mellitus: Epidemiology, Diagnosis, Management, and Research Imperatives With Insights From the United Arab Emirates and Global Contexts

**DOI:** 10.7759/cureus.105089

**Published:** 2026-03-12

**Authors:** Mathew Vadukoot Lazar, Arun C.S. Menon, Jibu Thomas

**Affiliations:** 1 Department of Gastroenterology, Lifecare Hospital, Abu Dhabi, ARE; 2 Department of Biotechnology, Karunya Institute of Technology and Sciences (Deemed to be University), Coimbatore, IND; 3 Department of Endocrinology, Aster Clinic, Bur Dubai (Aster Jubilee Medical Complex), Dubai, ARE

**Keywords:** diagnosis, epidemiology, exocrine pancreatic insufficiency, management, pancreatogenic diabetes, type 3c diabetes mellitus, united arab emirates

## Abstract

Type 3c diabetes mellitus (T3cDM), also known as pancreatogenic diabetes, is a form of secondary diabetes resulting from pancreatic disease and is frequently misclassified as type 2 DM (type 2 DM). A narrative review of peer-reviewed literature from international databases was conducted, with emphasis on the epidemiology, clinical differences, diagnostic complexities, and management of T3cDM, with a specific focus on the United Arab Emirates (UAE) and global contexts. T3cDM accounts for a notable proportion of global diabetes cases, yet it is underreported due to a lack of dedicated registries and frequent misclassification as type 2 diabetes. The UAE has one of the highest diabetes prevalence rates worldwide, yet T3cDM remains undercaptured. Unlike type 1 DM (T1DM) and T2DM, T3cDM is characterized by both endocrine and exocrine pancreatic insufficiency (EPI). The diagnosis requires evidence of pancreatic pathology, absent autoimmunity, and exocrine dysfunction. Management includes insulin therapy, pancreatic enzyme replacement therapy (PERT), and nutritional supplementation; recent advances include the role of incretin therapies, improved enzyme preparations, and regenerative medicines. Emerging approaches also include metabolomics for prediction and fecal microbiota transplantation. Increasing awareness, dedicating regional registries, and implementing multidisciplinary management strategies are urgently needed in the UAE and globally.

## Introduction and background

Type 3c diabetes mellitus (T3cDM) is a type of diabetes that arises from pancreatic disease, pancreatic resection, or injury, which affects the exocrine and endocrine functions of the pancreas [1,2]. Unlike autoimmune type 1 DM (T1DM) or insulin-resistant type 2 DM (T2DM), T3cDM results from structural and functional damage to the pancreas (e.g., chronic pancreatitis, pancreatic cancer, pancreatic trauma, cystic fibrosis, or pancreatectomy), disrupting pancreatic architecture and the incretin response [3,4]. The United Arab Emirates (UAE) provides a critical context for this discussion. Diabetes prevalence in the UAE is among the highest in the world. The International Diabetes Federation (2025) estimates the prevalence in the country to be almost 20.70% (1.25 million adults) [5], driven by urbanization, obesity, and genetic factors [6]. However, specific epidemiological data on T3cDM in the UAE remain limited because national registries currently classify diabetes primarily as type 1 or type 2 diabetes. Consequently, estimates of type 3 DM prevalence in the region are largely extrapolated from global epidemiological studies. In regions such as the Middle East, cultural fasting exacerbates glycemic instability in T3cDM, underscoring the need for tailored guidelines.T3cDM accounts for 5%-10% of diabetes worldwide and is not clearly documented globally due to underdiagnosis and misclassification [7]. T3cDM features unstable glucose control, malnutrition, and exocrine pancreatic insufficiency (EPI); misdiagnosis increases the risk of severe hypoglycemia, malnutrition, and pancreatic cancer, which is critical in high-prevalence areas like the UAE [8,9].

This article is a narrative review of the literature on T3cDM. A targeted literature search of peer-reviewed publications was conducted in PubMed, Scopus, and Google Scholar. Studies published between 2000 and 2025 were considered. Relevant international guidelines, review articles, and regional epidemiological studies addressing the epidemiology, diagnosis, and management of T3cDM were included. Key search terms included "Type 3c diabetes mellitus," "Pancreatogenic diabetes," "Post-pancreatitis diabetes mellitus," and "Exocrine pancreatic insufficiency."

## Review

Epidemiology

Incidence of Type 3c Diabetes in the UAE and Globally

T3cDM is estimated to account for 5%-10% of diabetes worldwide but reaches 25%-80% in chronic pancreatitis cohorts. This misclassification arises from T3cDM being classified as T2DM [2,10]. UAE registries omit T3cDM [6], capturing only T1DM/T2DM [11]. Post-pancreatitis DM (PPDM) is increasingly recognized as a part of T3cDM, with studies demonstrating a 7%-15% incidence after mild acute pancreatitis and higher rates following severe and recurrent episodes (Table 1) [12,13].

**Table 1:** Comparison of incidence and prevalence of T1DM, T2DM, and T3cDM (global and UAE estimates) Based on published literature [2,5,10,11] UAE: United Arab Emirates

Diabetes type	Global incidence/prevalence	UAE estimates	Key characteristics
Type 1 diabetes mellitus (T1DM)	Low incidence: 0.02%-0.3% globally; ~5%-10% of all diabetes cases	Rare, mostly in children/adolescents	Autoimmune β-cell destruction; insulin-dependent; no exocrine involvement
Type 2 diabetes mellitus (T2DM)	>90% of all diabetes cases worldwide; rising sharply with obesity and aging	~17% adult prevalence (among the highest globally)	Insulin resistance with relative insulin deficiency, associated with metabolic syndrome
Type 3c diabetes mellitus (T3cDM)	An estimated 5%-10% of all diabetes cases are often misclassified as T2DM	No official registry; underestimated; likely linked to high rates of pancreatitis and pancreatic disease	Secondary to pancreatic pathology (chronic pancreatitis, cancer, resection, trauma), both endocrine and exocrine dysfunction

Clinical distinctions

Differences From Type 1 and Type 2 Diabetes

T3cDM may be distinguished from T1DM and T2DM by its distinct pathophysiology, clinical features, and glycemic profiles [14]. Pathophysiological differences are different in each disease state. T1DM is an autoimmune disorder characterized by immune-mediated destruction of pancreatic β-cells and the resulting insulin deficiency. Pancreatic exocrine is preserved. T2DM results from insulin resistance and progressive loss of β-cell function, which are typically associated with obesity, physical inactivity, and genetic factors [15]. T3cDM is, on the contrary, a secondary diabetes that is developed due to pancreatic pathology (chronic pancreatitis, pancreatic cancer, or resection). It destroys both endocrine and exocrine tissues, leading to cellular dysfunction and reduced incretin response, thereby impairing glucose regulation. The clinical symptoms and complications vary in each case of diabetes. In younger people, T1DM is usually characterized by a sudden onset, and one of its first symptoms is diabetic ketoacidosis (DKA) [16]. T2DM is an insidious condition that occurs in middle-aged or older adults linked to obesity, high blood pressure, and problematic cholesterol. Patients with T3cDM, post pancreatic insult, are often malnourished and suffer from weight loss, steatorrhea, brittle glycemia, and fat-soluble vitamin deficiencies as a result of pancreatic exocrine insufficiency (PEI) [17]. In the UAE, vitamin D deficiency is associated with poorer outcomes [18]. Although glycemic patterns and exocrine involvement differ, both T1DM and T2DM are primarily associated with endocrine dysfunction; T3cDM presents a twofold problem involving both endocrine and exocrine dysfunction. This dual pathology underlies its unstable glycemic profile and the need to introduce therapies that address glucose management and nutritional deficiencies [19]. Increased pancreatic cancer risk in T3cDM necessitates vigilant surveillance [20].

Diagnosis

Diagnostic Criteria and Guidelines for Type 3c Diabetes

Diabetes classification: According to the World Health Organization (WHO) and the American Diabetes Association (ADA) [21], diabetes is classified into four categories: T1DM, T2DM, gestational diabetes (GDM), and other specific types. T3cDM is a type of diabetes in the latter category. However, there are no standardized diagnostic criteria for T3cDM globally, unlike those for T1DM and T2DM, making it challenging to identify and control. The best-known model is the list of criteria offered by Ewald and Bretzel [22], and according to which, a diagnosis of T3cDM requires existence of pancreatic exocrine disease (e.g., chronic pancreatitis, pancreatic cancer, or surgery), abnormal glucose metabolism that is consistent with diabetes, absence of autoimmune markers usually exhibited in T1DM, and objective evidence of pancreatic exocrine dysfunction, often assessed through tests like fecal elastase-1 or imaging modalities. These criteria emphasize the importance of documenting both endocrine and exocrine dysfunction when distinguishing from other forms of diabetes. Unlike T1DM, T3cDM is not an autoimmune disease, and diagnosis requires the absence of autoimmune markers such as glutamic acid decarboxylase (GAD) or islet cell antibodies.

Underdiagnosis of Type 3c Diabetes in the Arabic World

One of the key issues is the common misdiagnosis of T2DM due to the similarity of clinical presentation and the lack of national registries of pancreatic disease [23]. Moreover, there is a lack of diagnostic resources. Numerous medical centers lack fecal elastase testing, which is necessary to detect exocrine insufficiency, as well as more sophisticated pancreatic imaging modalities, such as magnetic resonance imaging (MRI) or endoscopic ultrasound. There is a lack of awareness among clinicians, leading to T3cDM being treated with standard T2DM alone, and the underlying exocrine pathology is not well addressed [24]. In the UAE, rapid urbanization strains specialized social services [13].

Impact on the Liver-Pancreas Axis in Diabetes Control

Another diagnostic difficulty is the interaction between the liver and the pancreas. Hepatic insulin resistance in T3cDM is associated with pancreatic exocrine dysfunction, which exacerbates glucose regulation [25]. Bilateral organ involvement complicates metabolic control compared with T1DM or T2DM, as it necessitates consideration of the hepatic and pancreatic roles in the development of dysglycemia. The inability to consider such interaction can lead to inefficient management and the increased risk of complications. Recent evidence links this axis to post-acute pancreatitis and diabetes, emphasizing the need for integrated assessments [2].

Relation of EPI With T3cDM

A defining feature of T3cDM is its strong association with PEI [2,25]. PEI causes malabsorption of nutrients, malnutrition, deficiency of fat-soluble vitamins (A, D, E, and K), weight loss, and steatorrhea. Such nutritional deficiencies also increase the instability of glycemic regulation, which leads to the so-called brittle diabetes so common among patients with T3cDM [26]. Notably, PEI is much more common in T3cDM (80%) than in T1DM (20%) or T2DM (30%), indicating its diagnostic value. Notably, PEI is more common in T3cDM than in T1DM and T2DM, indicating its diagnostic value [27].

Diagnostic work-up

T3cDM requires a multidisciplinary approach and evaluates endocrine and exocrine pancreatic function. A structured workup is essential for correct classification and management. The initial step is laboratory investigations. Fasting plasma glucose and HbA1c are standards for confirming hyperglycemia. C-peptide levels estimate residual β-cell function, and T1DM needs to be excluded by pancreatic autoantibody testing, such as GAD and insulinoma-associated antigen-2 (IA-2) [28]. Low C-peptide (<0.6 ng/mL) is common in advanced T3cDM [29]. The evaluation of exocrine insufficiency is crucial. Many people have recommended that the fecal elastase-1 test is a non-invasive, sensitive measure of exocrine function. However, it has recognized limitations, including possibly falsely low results in the presence of watery diarrhea, reduced sensitivity in mild EPI, and variability related to stool consistency, which should be considered when interpreting results. Seventy-two-hour fat absorption tests serve as supplementary evidence of steatorrhea or nutrient malabsorption in selected cases. Pancreatic computed tomography (CT) or MRIs are required to facilitate the visualization of structural alterations, such as atrophy, fibrosis, or calcification, which are typical of chronic pancreatitis. Endoscopic ultrasound may also be used if required [30]. In selected cases, further evaluation of pancreatic ductal anatomy using endoscopic retrograde cholangiopancreatography (ERCP) or magnetic resonance cholangiopancreatography (MRCP) may be required to clarify structural abnormalities contributing to pancreatogenic diabetes.

An integrated diagnostic algorithm-based on biochemical, functional, and imaging data-will ensure accurate diagnosis of T3cDM and reduce the risk of misdiagnosing T2DM (Table 2).

**Table 2:** Proposed diagnostic pathway for type 3c diabetes Based on published literature [2,22,25,28-30] CT: computed tomography; MRI: magnetic resonance imaging; GAD: glutamic acid decarboxylase; CGM: continuous glucose monitoring; HbA1c: glycated hemoglobin

Parameter	Type 1	Type 2	Type 3c
HbA1c	>6.5%	>6.5%	>6.5%
Fasting glucose	>126 mg/dL	>126 mg/dL	>126 mg/dL
Fasting C-peptide (0.5 to 2.0 ng/mL)	Very low: <0.2 ng/mL	Normal to high	Low to normal
Ketoacidosis	Common	Rare	Rare
Hypoglycemia (glucose < 69 mg/dL)	Common	Rare	Frequent
GAD/islet autoantibodies	Positive	Negative	Negative
Exocrine insufficiency (fecal elastase < 200 mcg/g)	Negative	Negative	Positive
CGM	Hyperglycemia and reactive hypoglycemia	Hyperglycemia	Alternate hyperglycemia and hypoglycemia
Pancreatic CT/MRI	Negative	Negative	Positive

Management and recent advances

Treatment Modifications Compared With Type 1 and Type 2 Diabetes

Diabetes management varies substantially, relying on the underlying pathophysiology. In T1DM (insulin dependence), complete insulin deficiency requires lifetime insulin administration. Oral hypoglycemics like metformin are used in T2DM, but insulin is used in the advanced disease. In patients with mild hyperglycemia and preserved β-cell function, metformin may be considered; however, evidence supporting its use in T3cDM is largely extrapolated from studies in type 2 diabetes, as randomized controlled trials specifically evaluating metformin in T3cDM remain limited. Insulin therapy is typically necessary in T3cDM, though the dosage is difficult to adjust because α-cell dysfunction negatively affects glucagon-mediated counter-regulation and predisposes to a greater risk of hypoglycemia [31]. In addition, reduced glucagon secretion resulting from pancreatic α-cell dysfunction may impair counter-regulatory responses to hypoglycemia, contributing to unstable glycemic control in patients with T3cDM. In the UAE, adjusting insulin regimens to accommodate cultural diets and fasting practices is essential [32]. Pancreatic enzyme replacement therapy (PERT) is essential for patients with PEI and is unique to T3cDM. Supplementing with enzymes enhances nutrient absorption, reduces cases of malnutrition, and normalizes fluctuating glycemic levels. This treatment is not required in T1DM and T2DM. While all three types of diabetes require an organized diet and physical exercise programs (lifestyle and nutritional changes), it is essential to note that T3cDM needs specific nutritional counseling, including supplementation with fat-soluble vitamins (A, D, E, and K) and individualized caloric intake to reduce malabsorption. In the UAE, multidisciplinary teams, including physicians, endocrinologists, gastroenterologists experienced in diabetic management, diabetic educators, dietitians, and podiatrists, manage such complex patients [33]. Continuous glucose monitoring (CGM) and insulin pumps are recommended for patients with unstable glycemic control [34]. The use of CGM and insulin pump therapy in T3cDM is largely extrapolated from studies in T1DM and brittle diabetes populations, as clinical trials specifically evaluating these technologies in T3cDM remain limited.

Hypoglycemic Agents and Recent Therapeutic Advances in T3cDM

Several advances have emerged in the management of T3cDM in recent years, but the evidence base is still limited. Incretin-based biotherapies, such as dipeptidyl peptidase-4 (DPP-4) and glucagon-like peptide (GLP-1) analogues, have demonstrated the potential to improve glycemic control by promoting insulin secretion and inhibiting glucagon secretion [35,36]. However, their use in T3cDM has been cautious, with some studies suggesting an association with pancreatitis. Hence, their use requires close monitoring in patients with recurrent pancreatitis [37]. Islet transplantation has been demonstrated to be efficacious in achieving glycemic stability in patients with brittle or refractory T3cDM. Improved immunosuppressive protocols and islet preservation techniques have enhanced outcomes, but cost and availability remain crucial challenges. The UAE is only in the first phase of exploring such strategies, but international trials show promise in specialized centers [38-41]. Emerging fields like metabolomics offer potential for predicting progression from diabetes to T3cDM, while fecal microbiota therapies show promise in modulating the gut-pancreatic axis [2]. Finally, PERT is also improving, driven by innovations that enhance nutrient absorption and by increased patient adoption. Recent long-acting, high-bioavailability formulations enhance digestion and help prevent complications of malnutrition, thereby stabilizing glycemic fluctuations (Table 3).

**Table 3:** Summary of the effects of hypoglycemic agents and recent therapeutic advances on glycemic variability and treatment in pancreatogenic diabetes mellitus Based on published literature [30,35-39,41,42] T3cDM: type 3c diabetes mellitus; DPP-4: dipeptidyl peptidase-4; GLP-1: glucagon-like peptide; SGLT-2: sodium-glucose cotransport inhibitors; CGM: continuous glucose monitoring; UAE: United Arab Emirates

Pharmacological group	Mechanism of action	Route of administration	Impact on variability	Treatment for T3cDM
Biguanides	Activate the AMPK signaling pathway and reduce insulin resistance	Oral	Neutral	First-line treatment
Sulfonylureas	Promote insulin release from pancreatic β-cells	Oral	Increase	Not recommended for use
Glinides	Promote insulin release from pancreatic β-cells	Oral	Unclear	Not recommended for use
Thiazolidinediones	Increase the sensitivity of target tissues	Oral	Decrease	Need more clinical trials to assess the benefits and risks
α-Glucosidase inhibitors	Prevent glucose absorption in the small intestine through competitive inhibition	Oral	Decrease	Not recommended for use
DPP-4 inhibitors	Incretins	Oral	Decrease	Require transparent clinical trials to demonstrate their safety and efficacy in patients with T3cDM
GLP-1 receptor agonists	Incretins	Subcutaneous	Decrease	Require transparent clinical trials that demonstrate their safety and effectiveness in patients with T3cDM
SGLT-2 inhibitors	Inhibit renal glucose and sodium reabsorption by blocking the action of SGLT-2	Oral	Decrease	Not recommended for use in patients with T3cDM until proven safe; more research is needed to explore their benefits and risks in T3cDM
Insulins	Regulate cellular uptake and utilization of glucose	Subcutaneous and intravenous	Decrease	Preferred regimen for patients who cannot achieve adequate blood glucose control with oral medications alone
Recent therapeutic advances in T3cDM				
Advance	Description	Evidence level	UAE relevance	
Incretin therapies (GLP-1/DPP-4)	Enhance insulin secretion; caution in pancreatitis history	Moderate	Monitoring in high-obesity populations	
Metabolomics prediction	Biomarkers for progression from prediabetes	Emerging	Potential for registries	
Fecal microbiota manipulation	Modulates the gut-pancreas axis for stability	Experimental	Research opportunity in diverse microbiomes	
Insulin pumps/CGM	For brittle diabetes, real-time adjustments	High	Available in the UAE specialist centers	

It should be noted that many pharmacological recommendations for T3cDM are based on expert consensus and pathophysiological considerations, as randomized clinical trials specifically evaluating glucose-lowering agents in populations remain limited.

Global Pancreatitis Etiologies Associated With Progression to T3cDM

Global etiologies of pancreatitis associated with progression to T3cDM vary worldwide, with alcohol abuse being the leading cause in many Western countries. Regional studies from the UAE have also highlighted the high burden of diabetes in diverse populations. At the same time, idiopathic, autoimmune, particularly IgG4-related disease, and genetic factors predominate in tropical regions, representing a recognized cause of pancreatogenic diabetes (Table 4).

**Table 4:** Global pancreatitis etiologies, progression to T3cDM Based on published literature [2,11,19,21,23,24,40,41,43] ERCP: endoscopy retrograde cholangiopancreatography; PERT: pancreatic exocrine replacement therapy; PEI: pancreatic exocrine insufficiency; IgG4: immunoglobulin G subclass-4; PCR: polymerase chain reaction; PTH: parathyroid hormone; MRCP: magnetic resonance cholangiopancreatography; HbA1c: glycated hemoglobin; CT: computed tomography; MRI: magnetic resonance imaging; T3cDM: type 3c diabetes mellitus

Pancreatic disease	Diagnostic evaluations	Time to diabetes	Monitoring & surveillance	Management (acute to long-term)
Biliary pancreatitis	Liver function tests, abdominal ultrasound, ERCP	2 months	HbA1c, glucose tolerance tests	Cholecystectomy, glycemic monitoring, insulin if needed, PERT for PEI
Alcoholic pancreatitis	History, amylase/lipase, CT pancreas	Months-years	Liver enzymes, glucose, and imaging for chronic pancreatitis	Alcohol cessation, insulin, PERT for PEI
Hypertriglyceridemic pancreatitis	Serum triglycerides, CT/MRI	Often in the early phase	Lipid panels, HbA1c	Lipid-lowering therapy, insulin for hyperglycemia, PERT
Drug-induced pancreatitis	Drug history, imaging	Variable	Medication review, glucose monitoring	Withdrawal of causative drug, standard diabetes care
Post-ERCP pancreatitis	Post-procedure amylase/lipase, imaging	Year	Routine diabetes screening post-procedure	Supportive care, insulin if β-cell loss occurs, PERT for PEI
Post-traumatic pancreatitis	CT, MRI, trauma history	Weeks-months	Regular glucose monitoring	Surgical management, insulin, nutritional support, PERT for PEI
Autoimmune pancreatitis	IgG4, imaging, biopsy	Probably months	Autoimmune markers, endocrine and exocrine monitoring	Steroids, immunosuppression, insulin, PERT for PEI
Infective pancreatitis (e.g., COVID-19)	PCR/serology, imaging, amylase/lipase	Probably months	Glucose, pancreatic imaging	Antiviral/supportive care, insulin, PERT if PEI
Hypercalcemic pancreatitis	Serum calcium, PTH levels, and imaging	Months	Calcium levels, glucose	Treat hypercalcemia, monitor for diabetes, and consider insulin if needed, PERT if required
Anatomical anomalies	MRCP, CT, ERCP	Months-years	Glucose monitoring & periodic imaging	Corrective surgery, insulin therapy, PERT if required

Research imperatives

Research Imperatives, Including Stem Cell Approaches

Research into stem cell and regenerative therapies offers a potential solution for the dual endocrine and exocrine dysfunction of T3cDM. Experimental results indicate that pluripotent stem cells can be differentiated into functional pancreatic β-cells and have the potential to restore insulin production. Early technologies are also exploring exocrine regeneration to enhance the digestive function [37,42]. Beyond regenerative medicine, there is an urgent need for region-specific registries in the UAE and the Middle East more broadly, as existing epidemiological data fail to capture T3cDM because it is often misdiagnosed as T2DM [43]. The development of biomarkers for early identification would improve the diagnostic tools and prompt intervention. Further, multicenter clinical trials specific to pancreatogenic diabetes are essential to establish optimal therapeutic protocols, especially in regions where the burden of pancreatitis-associated diabetes is increasing [44]. UAE-led trials could focus on metabolic syndromes prevalent in Gulf States [2].

Recommendations

Recommendations for Treatment of Type 3c Diabetes

The management of T3cDM should be multidisciplinary and integrated, addressing both endocrine and exocrine dysfunction. Controlling glucose levels is crucial; however, there is also a loss of both insulin-producing and glucagon-predominant cells, which necessitates close insulin titration to avoid hypoglycemia. In patients with EPI, PERT is necessary to enhance nutrient absorption, reduce steatorrhea, and stabilize glycemic fluctuations [45]. Nutritional counseling must focus on individualized diets, adequate protein intake, supplementation with fat-soluble vitamins, and small, frequent meals. Psychological support is also essential, as T3cDM patients are prone to high treatment burden and poor quality of life. A formalized clinical decision-making model must be used to assist physicians in making appropriate intervention decisions based on pancreatic function, comorbidities, and the risk of hypoglycemia [46].

Recommendations for Screening

**Figure 1:**
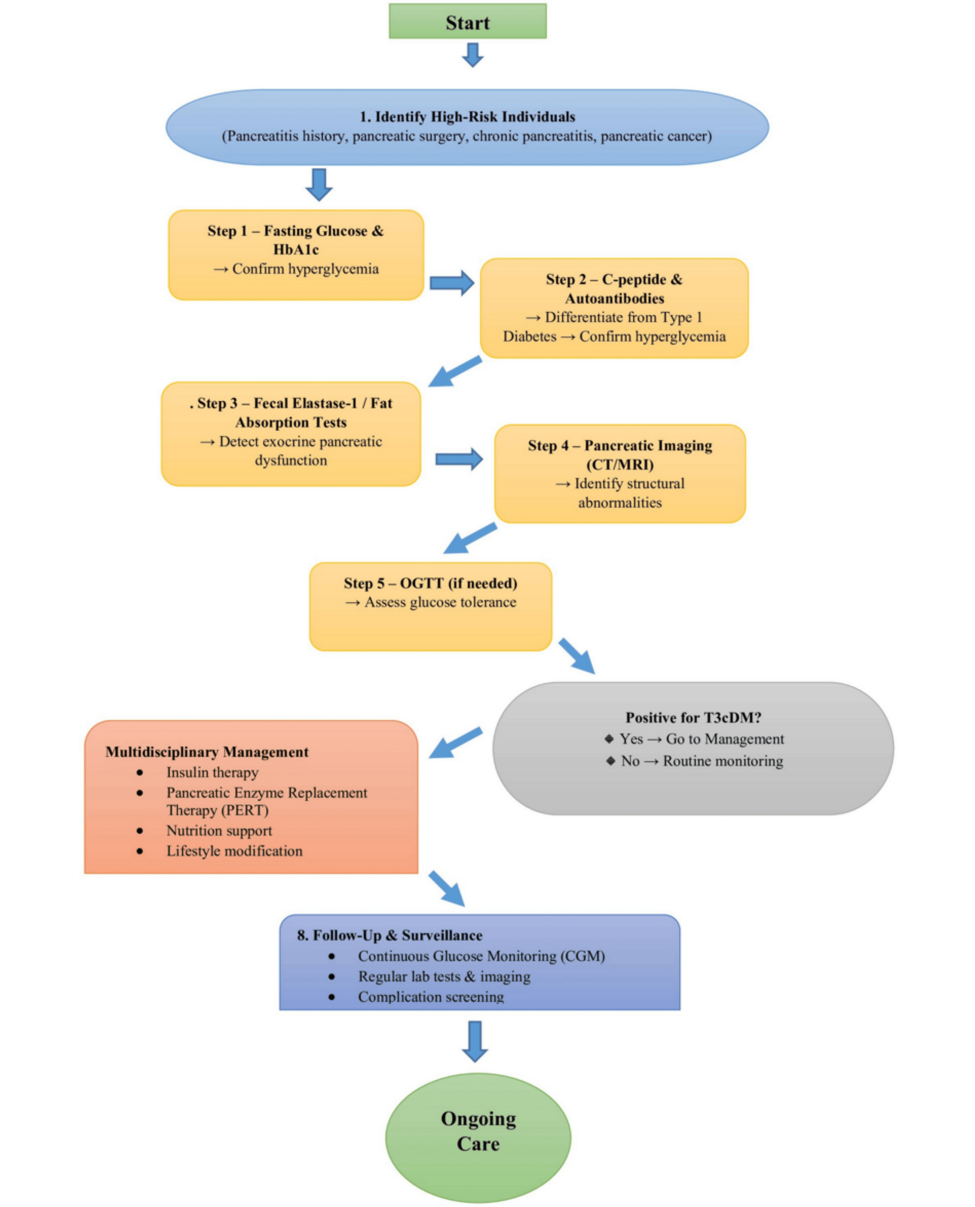
Flowchart for T3cDM: screening and management Author-generated schematic based on published literature and guideline recommendations [2,22,28,46] OGTT: oral glucose tolerance test; T3cDM: type 3c diabetes mellitus; HbA1c: glycated hemoglobin; CT: computed tomography; MRI: magnetic resonance imaging

As it is often underdiagnosed, screening interventions should focus on high-risk groups. Patients who have chronic pancreatitis or pancreatic cancer or who have undergone pancreatectomy are advised to monitor their glucose levels [47] regularly. Similarly, patients with acute pancreatitis should undergo systematic follow-up, as PPDM is a major cause of T3cDM. Testing should include HbA1c, C-peptide, and tests for exocrine insufficiency. Annual screening in high-risk cohorts in the UAE, as recommended by the Emirates Diabetes Society, could reduce misdiagnosis (Figure 1) [48].

This review has several limitations, including its narrative (non-systematic) design, potential for publication bias, limited region-specific data on T3cDM in the UAE, and variability in diagnostic criteria across published studies.

## Conclusions

T3cDM is underestimated, underdiagnosed, and poorly controlled as compared to T1DM and T2DM. Its unique pathophysiology involving failure of both endocrine and exocrine pancreas leads to unstable glycemia and nutritional difficulties with a high complication rate. Currently, global data gaps do not allow for an accurate estimate of the burden. As emphasized in this review, T3cDM differs fundamentally from T1DM and T2DM in epidemiology, pathophysiology, diagnostic strategy, and treatment. To increase awareness, it is necessary to establish registries that classify T3cDM separately, implement routine pancreatic exocrine function testing for patients with a history of pancreatic disease and long-term diabetes, and develop multidisciplinary models of care that include gastroenterology, endocrinology, nutrition, and primary care. The discovery of biomarkers, early post-pancreatitis screening, and the evaluation of integrated management methods should be prioritized in regional research agendas in the UAE and globally. These efforts will help improve patient outcomes and increase awareness of this neglected subtype of diabetes worldwide. This review positions T3cDM as a distinct clinical pharmacological entity requiring tailored therapeutic strategies that critically evaluate glucose-lowering therapies in the context of altered counter-regulation and metabolism, and highlight regional challenges such as fasting-related glycemic variability. It proposes an integrated diagnostic approach incorporating endocrine and exocrine management. This review highlights the epidemiological and clinical relevance of T3cDM, where prevalence is high but remains underrecognized due to limited registry data. It also defines priorities for dedicated clinical trials and registry-based research in pancreatogenic diabetes.
